# Editorial: Case reports in anxiety and stress

**DOI:** 10.3389/fpsyt.2023.1291083

**Published:** 2023-09-26

**Authors:** Ravi Philip Rajkumar

**Affiliations:** Department of Psychiatry, Jawaharlal Institute of Postgraduate Medical Education and Research, Puducherry, India

**Keywords:** anxiety disorders, post-traumatic stress disorder, comorbidity, prefrontal cortex, culture, trauma, neurostimulation

Anxiety disorders are a heterogenous group of mental illnesses characterized by fear or anxiety that is excessive and difficult to control, and which is associated with specific maladaptive behaviors (1, 2). Anxiety disorders are the most common form of mental illness, affecting almost 46 million people worldwide (3). The global burden of anxiety disorders has increased by around 50% in the past three decades, despite no significant change in their incidence (4). This is due to the chronic nature of these disorders: only about 60–70% of patients respond to treatment, and of these, up to two-thirds experience relapses (5, 6).

Anxiety disorders can often be triggered or exacerbated by stressful life events (7, 8). This is due to the effects of both acute and chronic stress on specific neurotransmitters, neurohormones, and the expression of specific genes (9–11). Stress-related disorders such as post-traumatic stress disorder (PTSD) are highly comorbid with anxiety disorders (12). These groups of disorders share common vulnerability genes and molecular pathways (13), and exposure to traumatic stressors can trigger not just PTSD, but any of the anxiety disorders (14). This was reflected in earlier classifications that placed anxiety and stress-related disorders in the same general category (15). Though recent research has identified key differences between them (16), their overlap remains both conceptually and clinically significant (17).

From a historical perspective, anxiety and stress-related disorders have often been viewed as “neurotic disorders”, arising from unresolved psychological conflicts, and requiring resolution through psychodynamic therapies (18). Today, these disorders are viewed as a heterogeneous but interrelated group of conditions, arising from complex interactions between genetic vulnerability, early life experiences, and subsequent stressors (19). The “final common pathway” for these disorders probably involves mislabeling of environmental stimuli as threats (alarms), misinterpretation of these threats and their consequences (beliefs), and subsequent maladaptive behaviors (coping). The neuroanatomical substrates of these events include the prefrontal cortices, amygdala, hippocampus, insula, and basal ganglia (16, 20).

The current Research Topic presents seven papers examining the epidemiology, diagnosis, and management of anxiety and stress-related disorders. These reports reflect the diversity of this group of conditions, the clinical challenges faced in treating them, and their relationship to both individual and collective forms of stress and trauma.

Hallucinations are typically associated with psychotic disorders, but they can be a manifestation of significant anxiety or stress (21, 22). Jiang et al. report the case of an elderly woman with olfactory hallucinations associated with generalized anxiety disorder. No evidence of a psychotic, neurological or nasopharyngeal disorder was found. The patient's hallucinations resolved with pharmacological management of the anxiety disorder.

Culture can significantly shape the presentation of anxiety disorders by modifying interpretations of specific situations, the health-related consequences attached to them, and the specific illnesses they may represent. This can lead to unusual symptom presentations, particularly in Asian and African cultures (23, 24). Religious and spiritual beliefs and practices can also influence anxiety symptomatology (25). Khoe and Gudi describe the case of a patient with recurrent trance and possession episodes, associated with auditory and visual hallucinations. These symptoms were found to be panic attack equivalents, shaped by religious beliefs and guilt related to a past life event. As in the first case, the patient responded to pharmacological treatment for panic disorders. These cases highlight the challenges involved in diagnosing anxiety disorders across cultures.

Collective forms of trauma are associated with significant increases in anxiety and stress-related disorders (26–28). Limone et al. systematically reviewed the literature on anxiety and stress in students in relation to the COVID-19 pandemic and the Russia-Ukraine war. They found high levels of both anxiety symptoms (14–89%) and stress (28–56%) in this population. Risk factors associated with these symptoms included female gender, course of study, social isolation, prior physical or mental illness, and the impact of these disasters on students' families and friends. Kurapov et al. reviewed studies from the first 6 months of the Russia-Ukraine war, and found high levels of both anxiety (36%) and stress (70%) in the Ukrainian general population. Of those with symptoms of anxiety, over one third had severe symptoms suggestive of an anxiety disorder. Risk factors in this population included female gender, older age, financial difficulties, unemployment, forced relocation, and direct experience of war-related trauma. These findings highlight the need for access to adequate mental health care and psychosocial support in conflict and disaster settings.

Anxiety disorders can also be caused by general medical conditions. These “secondary” anxiety disorders should be suspected in patients with atypical symptom presentations (29, 30). Geng et al. describe a patient with Cushing's disease, initially misdiagnosed as generalized anxiety disorder. Zhai et al. describe a patient whose anxiety symptoms were found to be related to a patent foramen ovale. In the first of these cases, the patient experienced a severe adverse reaction to standard doses of an SRI. These cases highlight the biological links between anxiety and factors such as cortisol levels (31) or central perception of suffocation (32), the importance of identifying and treating the underlying medical disorder, and the need for caution when prescribing psychotropics in medically unstable patients.

Response to standard pharmacological or psychological treatments in anxiety and stress-related disorders is often unsatisfactory (5, 6, 33). Non-invasive neurostimulation methods, such as repetitive transcranial magnetic stimulation (rTMS) and transcranial direct current stimulation (tDCS) have shown preliminary evidence of efficacy in anxiety disorders and PTSD (34). Chang et al. describe the successful use of accelerated theta-burst rTMS, applied over the dorsolateral prefrontal cortices (DLPFC), in a patient with treatment-resistant PTSD occurring in the context of emotional and physical abuse. This result is consistent with the existing literature, and highlights the importance of the DLPFC as a key component of the “executive control” network involved in anxiety and stress responses (35, 36).

Overall, the papers included in this Research Topic provide a snapshot of the biological, social, and cultural dimensions of anxiety and stress-related disorders, and will be of interest to clinicians, researchers and public health experts.

## Author contributions

RR: Conceptualization, Writing—original draft, Writing—review and editing.
